# Editorial: Neurobiology of *Drosophila*: the 19th NeuroFly-2022 meeting

**DOI:** 10.3389/fphys.2023.1237065

**Published:** 2023-06-15

**Authors:** Jean-René Martin, Abhishek Chatterjee, Iris Salecker

**Affiliations:** ^1^ Institut des Neurosciences Paris-Saclay, CNRS, Saclay, France; ^2^ Institut d’Écologie et des Sciences de l’Environnement de Paris (iEES-Paris), INRAE, Versailles, France; ^3^ Institut de Biologie de l’École Normale Supérieure (IBENS), ENS, CNRS, INSERM, Université PSL, Paris, France

**Keywords:** *Drosophila*, neurobiology, neural circuit function and assembly, neurons, glia, behavior, sensory systems, neurodevelopmental and neurodegenerative diseases

**FIGURE 1:**
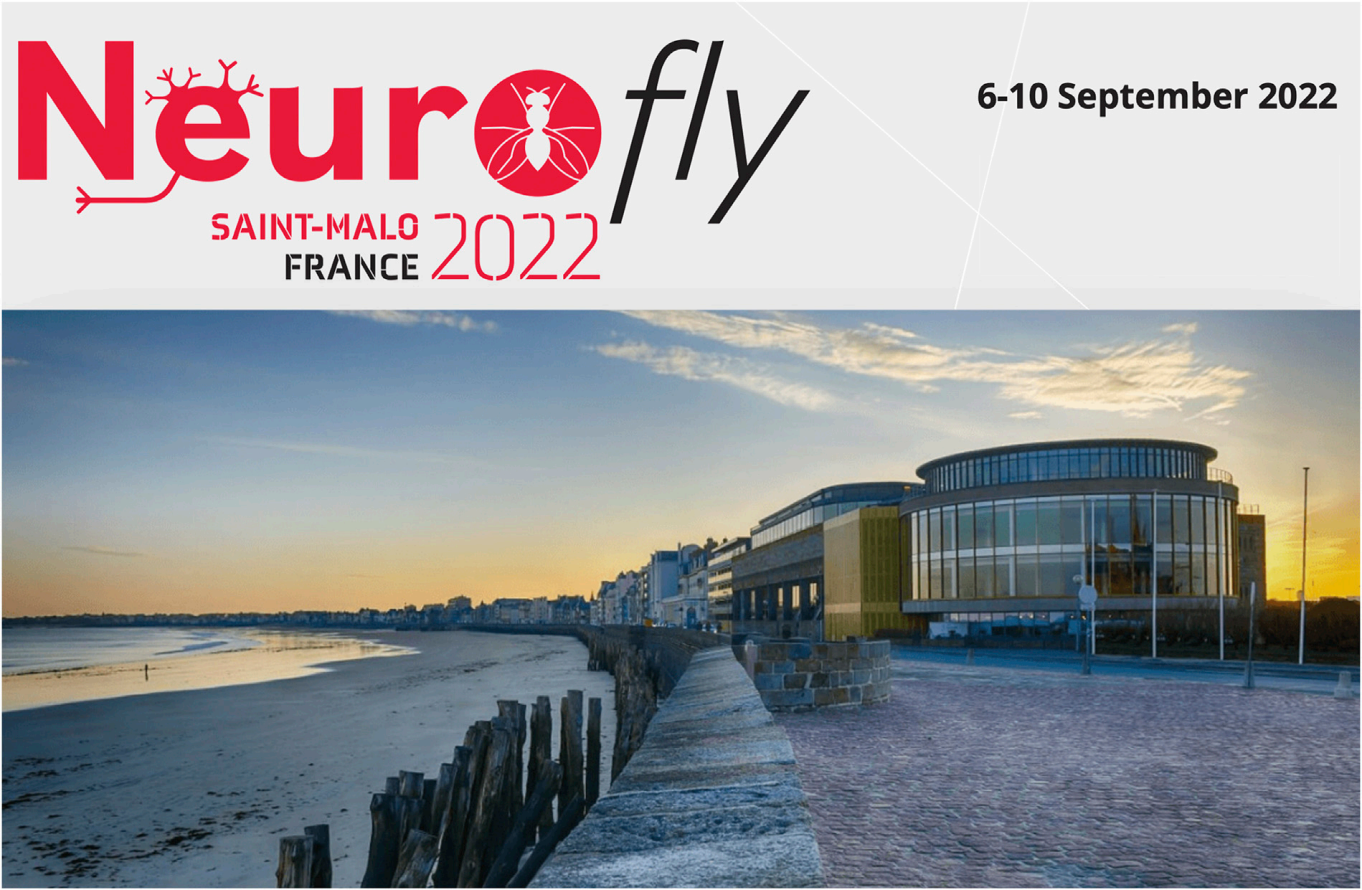
The conference center Palais du Grand Large in Saint Malo, Brittany, France.

In September 2022, close to 375 neurobiologists from 35 different countries came together in Saint-Malo (France) to attend the 19th European *Drosophila* Neurobiology Conference (Figure 1). This biennial meeting is organized by scientists and takes place in different European cities, each time offering a distinct thematic flavor and personal touch. The strong participation highlighted both the vitality of the *Drosophila* neurobiology community and the need to meet colleagues in person to share our science and convivial moments.

Sessions of NeuroFly-2022 were organized around the themes of Emerging Technologies, Brain Homeostasis and Metabolism, Brain Evolution and Ecology, Neural Development, Neural Circuits and Synapses, Brain Disorders, Sensory Systems and Behavior. Yet, sessions had no boundaries as thematic threads were taken up repeatedly. Assisted by web-based resources, such as “Virtual Fly Brain” described by Court et al., our knowledge of developing and adult neural circuits has become remarkably detailed. This, combined with cutting-edge genetic and other technologies, allowed the community to leap forward by addressing questions in an integrated manner–bridging connectomics and behavior, single cell transcriptomics and evolution of sensory systems, neuron and glial biology united in the context of nervous system development and function.

Starting with the latest technological innovations, we learned that automated functional brain imaging now enables long-term recording of neural activity in the adult brain *in situ* to unravel the logic underlying locomotion patterns and awake/sleep cycles with single-cell resolution (Flores-Valle et al., 2022). Moreover, large-scale genomics approaches, including single-cell RNAseq transcriptomics, help to robustly assign molecular signatures to developing and adult neuronal subtypes, glia or hemocytes, and to embark on evolutionary comparisons (Konstantinides et al., 2022; Sakr et al., 2022).

Throughout the meeting, presentations explored the mechanisms underlying learning and memory from different angles. Subcellular mechanisms controlling the transport and synaptic targeting of specific mRNAs shape long-term memory formation (Bauer et al., 2023). Brain homeostasis and metabolism studies highlighted the crucial control of energy flux in mushroom bodies by cortex glia for memory formation (Silva et al., 2022). Multisensory input results in improved memory retrieval through cross-modal binding of selective neurons via serotonergic microcircuits (Okray et al., 2023).

*Drosophila* revealed its remarkable potential for investigating the molecular and cellular basis of neurodevelopmental and neurodegenerative disorders. Abaquita et al. thus began to untangle the dual neurotoxic and neuroprotective actions of enzymes in oxidative stress-dependent neurodegeneration. While still awaiting compelling assertion of causality, circadian and sleep syndromes serve as early red flags and proxy of neurodegenerative disease progress. Pinpointing responsible genetic lesions made it possible to design combined pharmacological and behavioral interventions, such as sleep restriction, and to bring these notions to the clinic (Coll-Tane et al., 2021).

To unravel the mechanisms underlying circuit assembly, multi-scale explorations remain key. This is illustrated by Lin’s comprehensive review of mushroom body development. Structure-function analyses of the cell surface molecules Sidestep and Beaten path by Heymann et al. uncovered an unexpected level of functionality during motoneuron development that includes proteolytical cleavage and nuclear import. The insect brain has been considered as stereotypic and hard-wired. However, during the meeting the notion stood out that the process of circuit assembly is highly dynamic and plastic. Live imaging combined with genetic and temperature manipulations in the visual system revealed two important principles ensuring that synapses are formed with correct postsynaptic partners based on spatiotemporal availability: 1) pattern formation through growth cone self-organization, and 2) filopodial kinetics influencing the duration of interactions (Kiral et al., 2021; Wit and Hiesinger, 2023). Moreover, the molecular mechanisms underlying structural plasticity are coming into reach. These include *Drosophila* neurotrophins, acting through Toll-like receptors and Trk-like kinase-less receptors (Kekkons) to control survival, arborization patterns and synapse numbers (Li et al., 2020). Coulson et al. added strong evidence for temporally restricted developmental windows of heightened plasticity, *i.e.*, critical periods, that are essential for the formation of robust functional neural circuits.

Moreover, glia shared the stage with neurons. Investigations of different glial classes revealed that these participate in the remodeling of different neuron subtypes involving the chemokine-like protein Orion (Boulanger et al., 2021; Boulanger and Dura, 2022). Studies of peripheral wrapping glia uncovered intriguing links between glial-dependent clustering of voltage-gated ion channels and the evolution of myelination (Rey et al., 2023). Shaping higher-order brain functions, astrocytic D-serine gliotransmission promotes thirst-directed behaviors (Park et al., 2022).

*Drosophila* shows a sophisticated behavioral repertoire in response to sensory inputs. Connectome approaches identified novel visual projection neurons that transmit parallel information to the central complex, mediating polarized sky light navigation (Kind et al., 2021). Searching for the mechanisms underlying magnetoreception, non-canonical, non-Cryptochrome (CRY)-dependent radical pairs were shown to elicit cellular magnetic-field responses in flies (Bradlaugh et al., 2023). Moreover, mechanosensory neurons that control grooming of head regions establish a somatotopic brain map (Eichler et al., 2023). Neurogenetic, imaging and behavioral studies identified mushroom body neurons that decode the value rather than the specificity of an odor (Das Chakraborty et al., 2022; Villar et al., 2022), whereas Mohamed et al. uncovered neurons implicated in intensity-dependent odor discrimination learning.

Presentations embarked on novel, unexpected research topics and shifted textbook paradigms. We learned that fly brains, as their vertebrate counterparts, develop left-right asymmetries (Lapraz et al., 2023). Bengochea and Hassan reported that flies are amenable to studying the mechanisms underlying numerical discrimination. Live imaging and an elegant photoablation method of Mangione et al. revealed that in addition to the classical sensory organ precursor divisions, a fifth epidermal cell is co-opted into tactile bristles that contributes to mechanosensory function (Mangione et al., 2023).

Together, the many remarkable discoveries presented at this meeting truly revealed the inventive capacity of *Drosophila* research to tackle complex mechanisms across scales. The scientific content of NeuroFly-2022 was outstanding. Plenary and selected talks were followed by myriads of questions, and poster sessions were buzzing. In the continuity of this stimulating meeting, eight manuscripts–mentioned throughout this Editorial–have been published in this Research Topic. To conclude, we are delighted to see that the NeuroFly meeting tradition is stronger than ever: thus, let’s meet again in Birmingham, United Kingdom, in 2024!
